# Renal Lithiasis

**Published:** 1913-11

**Authors:** 


Renal Lithiasis. J. Bentley Squire, M. D., *Am. Jou. Surg.,* April, 1913. He divides calculi into primary and secondary according to probability of origin. The term secondary applied to those which can be definitely proven to be dependent for their causation upon bacterial invasion of the kidney. The causation of primary stones is still a matter of controversy and he cites a number of theories, but says it must be left to the experimental laboratory for final solution.

In regard to early diagnosis, attention in many cases is first directed to urinary trouble by albumin being found in course of insurance examination for life policy. The classic symptoms-hematuria, renal colic and fixed pain being so often absent as to be negligible, so more minor symptoms must first attract to possible seat of disease.

He emphasizes the necessity of making radiographs of both kidneys, citing a number of cases where symptoms all pointed to one kidney where radiograph showed calculi in both kidneys. In operating upon these cases the rule is to operate upon the less diseased kidney first and the other at a subsequent date. The newer renal function tests make it comparatively simple to arrive at a definite conclusion as to which kidney is the less damaged, also, it is better to rely upon this rather than the size and number of calculi as shown in radiograph.

When operating for the removal of very small calculi he overcomes the difficulty of locating the stones by means of a small portable X-ray apparatus with right-angled vision fluoroscope with which he examines the kidney after it has been exposed and brought out on the loin. This requires but a moment, and in doubtful cases is of the greatest value.

